# Postoperative Radiotherapy for Glioma: Improved Delineation of the Clinical Target Volume Using the Geodesic Distance Calculation

**DOI:** 10.1371/journal.pone.0098616

**Published:** 2014-06-04

**Authors:** DanFang Yan, SenXiang Yan, ZhongJie Lu, Cong Xie, Wei Chen, Xing Xu, Xinke Li, Haogang Yu, Xinli Zhu, LingYan Zheng

**Affiliations:** 1 Department of Radiation Oncology, The First Affiliated Hospital, College of Medicine, Zhejiang University, Hangzhou, Zhejiang, PR China; 2 State Key Lab of CAD & CG, Zhejiang University, Hangzhou, PR China; The George Washington University, United States of America

## Abstract

**Objects:**

To introduce a new method for generating the clinical target volume (CTV) from gross tumor volume (GTV) using the geodesic distance calculation for glioma.

**Methods:**

One glioblastoma patient was enrolled. The GTV and natural barriers were contoured on each slice of the computer tomography (CT) simulation images. Then, a graphic processing unit based on a parallel Euclidean distance transform was used to generate the CTV considering natural barriers. Three-dimensional (3D) visualization technique was applied to show the delineation results. Speed of operation and precision were compared between this new delineation method and the traditional method.

**Results:**

In considering spatial barriers, the shortest distance from the point sheltered from these barriers equals the sum of the distance along the shortest path between the two points; this consists of several segments and evades the spatial barriers, rather than being the direct Euclidean distance between two points. The CTV was generated irregularly rather than as a spherical shape. The time required to generate the CTV was greatly reduced. Moreover, this new method improved inter- and intra-observer variability in defining the CTV.

**Conclusions:**

Compared with the traditional CTV delineation, this new method using geodesic distance calculation not only greatly shortens the time to modify the CTV, but also has better reproducibility.

## Introduction

Postoperative adjuvant radiotherapy has become the standard of care in the management of gliomas [1]. Unlike many other malignancies, the main pattern of treatment failure in gliomas is local recurrence, which has prompted clinicians either to boost the radiation dose or to optimize the radiation target so as to improve local control of the disease. Encouraging results have been reported using intensity-modulated radiation therapy (IMRT) for gliomas [2], [3]. With IMRT, the dose distribution can better fit the target volumes than conformal radiotherapy. As a result, accuracy and consistency in target contouring should be highlighted when using IMRT to yield dosimetric benefits and to reduce inter- and intra-observer variability.

Definition of the target volume of gliomas is governed by rules [4]. Take glioblastoma multiforme (GBM), for example, in the European Organization for Research and Treatment of Cancer (EORTC) criteria, the gross tumor volume (GTV) is defined in terms of the region of enhancement on preoperative or postoperative CT/MRI scans. The clinical target volume (CTV) is defined as the GTV plus a 2 to 3 cm margin to allow for microscopic spread. In spite of this guideline, variations in target volume delineation still exist. The CTV is considered to be the most important factor contributing to deviations in treatment planning. Variations in CTV delineation could be generated either by a number of observers (inter-observer) or even a single observer (intra-observer) at different times in one patient following the same criteria [5].

There are two basic methods used for defining distance: the Euclidean distance and the geodesic distance [6]. The Euclidean distance refers to the linear distance between two points in space, while the geodesic distance is the shortest distance between two points along a curved surface. Traditional calculation of the CTV, which is used in the currently available treatment-planning system (TPS), directly applies Euclidean distance with simple diffusion, while ignoring natural spatial barriers. The CTV is formed as a spherical shape when adding a 2 or 3 cm margin to the GTV. However, glioma cells infiltrate the surrounding tissues irregularly rather than in the form of a spherical shape [7]. Natural barriers, such as the skull, cerebral falx, tentorium cerebelli and ventricles could prevent glioma cells from infiltrating in some directions. In addition, migrating tumor cells could detour to the contralateral hemisphere through structures such as the corpus callosum. Thus, the above barriers must be considered when performing CTV expansions. Clinically, physicians undertake a great number of manual modifications on each slice of the CT simulation at a later stage to allow for the barriers, so that generation of the CTV from the GTV is a ‘geodesic’ approach. This is definitely a time-consuming process and of poor reproducibility. It is also the main reason for the inter- or intra-observer variability in the delineation of the CTV.

Herein, we introduce a new method for the calculation of geodesic distance using a combination of medical imaging, three-dimensional (3D) geometric calculation and medical visualization analysis with the objective of improving the accuracy and consistency of CTV delineation.

## Materials and Methods

The study was approved by the Ethics Committee of the First Affiliated Hospital of College of Medicine at Zhejiang University and written informed consent was obtained from the single GBM patient enrolled. Prior to radiotherapy, a CT simulation was performed using a dedicated CT scanner (Sensation Open: Siemens, Munich, Germany). Images were transferred to the TPS (Eclipse^™^: Varian, Palo Alto, CA, USA), and the GTV and natural barriers such as the skull and cerebral falx were contoured on each CT slice.

**Figure 1:**
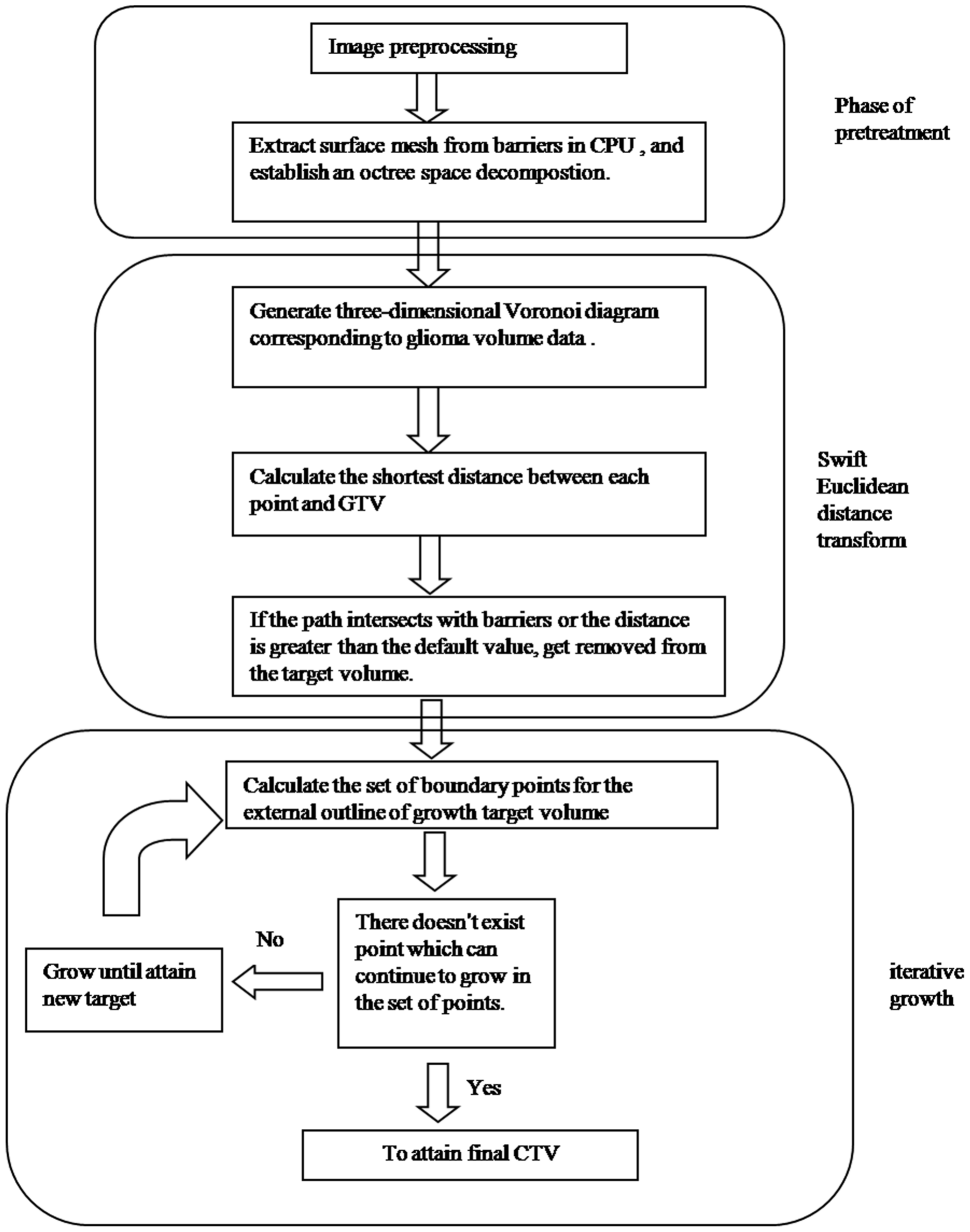
The flow process diagram regarding calculation of the CTV. *CTV: clinical target volume.*

CTV expansion can be considered as the calculation of a 3D distance field from the GTV. The GTV and natural barriers can be shown in terms of triangular tiles or regular volume data, which determine the different methods of calculation. Because of the existence of barriers in the space field, it is necessary to perform multiple sheltering judgments whenever the shortest distance from one voxel to the GTV is being calculated. In the present study, we performed geodesic distance calculations to generate the CTV using a fast parallel implementation of the 3D Euclidean distance transform, taking into account the impact of spatial barriers using a multiple iterative growth method. Furthermore, we sped up the intersection calculation by means of space decomposition based on the octree method. **Figure 1** shows the detailed process of geodesic distance calculation.

**Figure 2:**
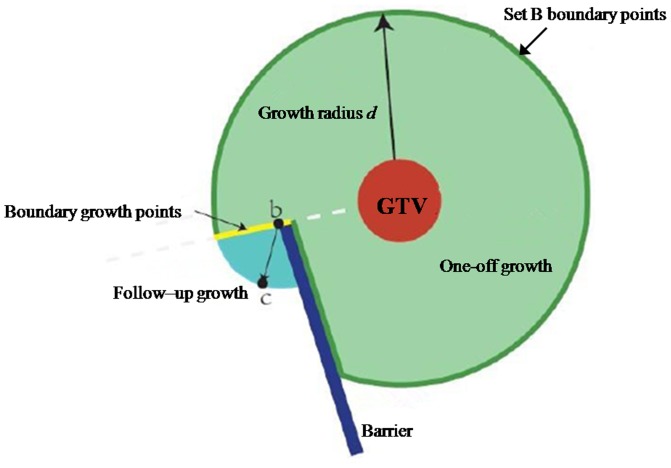
Schematic diagram of growth iterations.

After the 3D Voronoi diagram had been obtained and the spatial octree for the barriers constructed, we obtained the final target volume by calculation of the multiple iterative growths. First, the CTV was generated using the default external diffusion value (i.e. the growth radius, *d*) based on the GTV. In the first growth iteration, the system calculated the shortest distance in space from the GTV for each point based on the 3D Voronoi diagram to attain the corresponding segment *L*. If the distance was longer than *d*, the corresponding point was removed from the growth target volume. If the distance was shorter than *d*, it was necessary to judge if the segment *L* intersected with the barriers. If the segment *L* intersected with the barriers, the point was similarly removed from the target volume. The green area in **Figure 2** represents the results that were obtained. For each point in space, if *ds* was denoted as the cumulative shortest distance between the point and the GTV, then *ds* for each point in the target volume was the shortest Euclidean distance away from the GTV in the first growth iteration.

Based on the previous growth iterations, the distance field for the area sheltered by the barriers was recalculated for the following growth iterations. As for point *a* from the previous growth target volume in the traversal space of the system, if its six domain contained points that did not belong to the target volume point *a*, they were added to set *B* of boundary points (shown by the deep green outline in **Fig. 2**). As for each point in set *B*, if point *b* did not border upon the barriers and its *ds* value was smaller than *d*, this point underwent follow-up growth as a boundary growth point. As shown by the yellow segment in **Figure 2**, the value of the follow-up growth radius *d'* was equal to *d* minus *ds* for point *b*. In the course of the follow-up growth evaluation, the growth space was limited to a cube with *b* as the central point. Thus, the distance from *b* to each face of the cube amounted to *d'*. If the distance from a certain point *c* to *b* in the cube was <*d'*, and if the straight line connecting the two points did not intersect with the barriers, it was necessary to calculate the sum (*dc*) of *ds* for point *b* and the distance between point *c* and point *b*. Otherwise, the point *c* was removed from the target volume. As for the calculated value of *dc*, if it was smaller than the present value of *ds* for point *c*, it should be updated and added to the target volume. After multiple follow-up growth iterations, the cumulated Euclidean distance *ds* from each boundary point to GTV was equal to *d*.

To test the usefulness of this new method, five radiation oncologists with at least 5 years’ experience were asked to delineate the CTV based on the same GTV using the TPS (EclipseTM: Varian). The CTV was defined as the GTV plus a 2.5 cm margin. Using the traditional method, after automatic and isotropic expansion as a regular spherical shape, the CTV was manually modified to account for the skull and cerebral falx. Then one single physician was also asked to delineate the CTV on 5 different days. Using the current new method, the five physicians and one single physician on 5 different days were asked to delineate the skull and cerebral falx; then the CTV was automatically generated from the GTV. The inter- and intra-observer variability in the delineated volume and the conformity index (CI) were compared. The delineated volume and the CI were calculated using TPS software. The CI of the five delineated structures was defined as the ratio of the overlapping volume and the encompassing total delineated volume. A CI of 1 indicated perfectly overlapping volumes. A CI of 0.5 indicated that the observers agreed regarding 50% of the total delineated volume [8]. Moreover, the time taken to generate the final CTV was compared between this new approach and the traditional one.

## Results and Discussion

**Figure 3:**
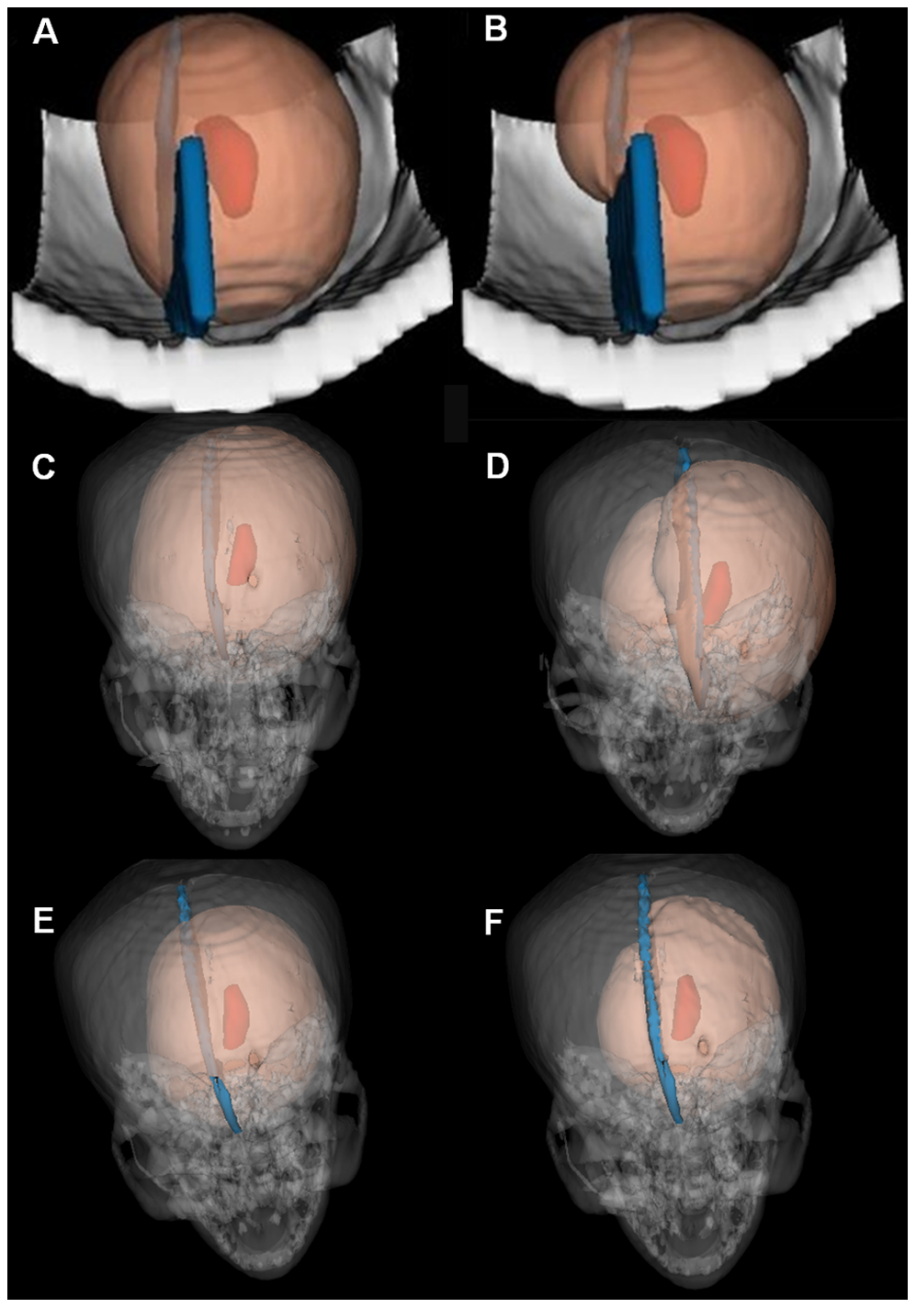
Two-dimensional images (A–B) and 3D visualization (C–F) results from the CTV delineation in different situations. (A) The model of isotropic external expansion using the direct Euclidean distance calculation; (B) The model of anisotropic external expansion involving the use of the geodesic distance calculation to automatically cross the cerebral falx; (C) Generating the CTV without taking into consideration the cerebral falx or skull; (D) Considering the cerebral falx but not the skull; (E) Considering the skull but not the cerebral falx; (F) Considering both the skull and the cerebral falx. *The deep red area represents the GTV, the blue area the cerebral falx, the grey area the skull, and the pink area the CTV. GTV: gross tumor volume; CTV: clinical target volume.*

In the current study involving GBM, the GTV was tracked as feature points when the 3D Voronoi diagram was computed, and the skull and cerebral falx were tracked as barriers when the final CTV was calculated automatically. **Figure 3** **(A) and (B)** shows the models that were used to compare the CTV results obtained using direct Euclidean distance expansion and geodesic distance calculation; a regular spherical-shaped CTV could be seen in the former approach, and considerable manual effort was required at a later stage to modify the CTV. In contrast, using the geodesic distance calculation that takes the corpus callosum and cerebral flax into consideration, the CTV was generated much more reliably. **Figure 3** **(C–E)** shows the 3D visualization results from the CT simulation when the CTV was generated automatically using the geodesic distance calculation, when taking into consideration nothing, the cerebral falx, the skull. **Figure 3** **(F)** shows the CTV that was obtained using the current geodesic distance calculation method to automatically cross the cerebral falx and the deselected area beyond the skull.

**Figure 4:**
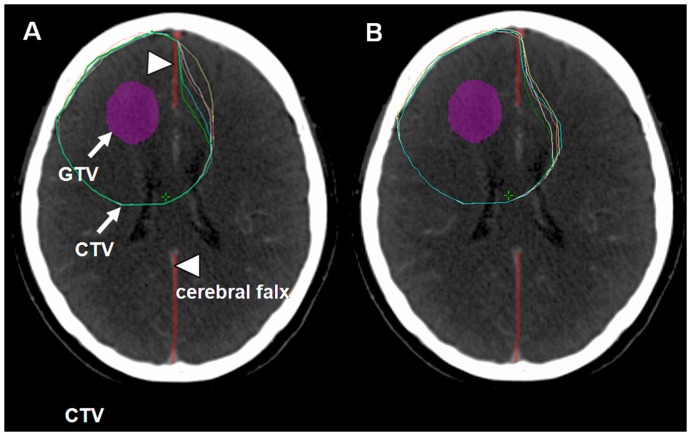
Inter- and intra-observer variability. (A) Inter-observer variability in the multiple contours from five observers; (B) Intra-observer variability involving a single physician at different times.

**Figure 4** **(A)** demonstrates the disagreement (inter-observer variability) regarding the CTV volume and shape among the five physicians using the traditional CTV delineation. Furthermore, inconsistency (intra-observer variability) involving one single physician on 5 different days could also be found, as shown in **Figure 4(B)**. **Table 1** shows the inter- and intra-observer variability in the delineated volume using the traditional method. No modification was required using the current new method, and the only disagreement between physicians came from the delineation of the skull and cerebral falx; the CI was almost 100%. The study demonstrated that the current new method had great reproducibility.

**Table 1:** Inter- and intra-observer variability in the delineated volume and conformity index using the traditional method for generating the clinical target volume.

	Volume (cm3)						Conformity index
	Contour 1	Contour 2	Contour 3	Contour 4	Contour 5	Volume mean ±std	
Inter-observer	**234.04**	**220.93**	239.93	299.80	310.05	260.95±18.29	63.7%
Intra-observer	220.93	217.66	215.43	222.97	225.62	220.52±1.82	87.5%

As shown in **Table 2**, the new method also greatly reduced the time required for the physician to modify the CTV, which was 0.98, 1.04, and 1.23 seconds, for different CTV expansion radii. In contrast, using the traditional method, further modification by oncologists was time consuming, taking approximately 5, 6, and 8 minutes for different CTV expansion radii.

**Table 2:** Comparison of the time required to generate the clinical target volume between the newly developed method and the traditional method involving manual modification.

Expansion radius (mm*)	15	20	25
New method (s**)	0.98	1.04	1.23
Manual modification (s)	≈300	≈360	≈480

*millimeter;

**second.

If we take a bird's-eye view of postoperative radiotherapy for glioma over the past decades, great progress has been achieved, ranging from target volume and radiation dose delivery to physical technology. With regard to physical technology, IMRT has been shown to improve radiation dose distribution in the target volume, as well as to reduce the dose exposure of normal tissues compared with conventional radiotherapy [9]. Some encouraging outcomes have been achieved in clinical studies involving IMRT, which undoubtedly will be the mainstay of radiotherapy technology for the treatment of gliomas in the future. However, further improvement in the accuracy of target volume delineation is required, especially with regard to CTV delineation [10], because variability in CTV delineation is considered to be the most important factor contributing to the global uncertainty in planning definition.

In recent years, greater attention has been focused on target volume delineation. The use of CT and MR registered imaging has been reported to reduce inter-observer variability in target volume delineation regarding postoperative irradiation of high-grade gliomas [11]. Some studies have used diffusion tensor imaging (DTI) to improve the delineation of target volumes in glioma radiotherapy, and have demonstrated that DTI can improve and individualize margin delineation and reduce the size of the CTV, thus permitting modest dose escalation [12], [13]. However, the delineation of margins in these studies still requires further manual modification because of the presence of anatomical regions such as the skull, cerebral falx, and tentorium cerebelli where tumor spread is unlikely. There is no doubt that contour variability depends on the composition of the manual modification, and the more organs or structures that need to be taken into account, the greater the variability observed. Further manual modification involves a considerable number of subjective components that can vary between oncologists or centers even when they are based on the same rules. In the present study, we preliminarily researched the possibility of automatically generating the CTV using the geodesic distance calculation involving the parallel Euclidean distance transformation method so as to reduce the manual work required and inter- or intra-observer variability. Using this approach, there was only small variability stemming mainly from the contouring of the anatomical barriers, which showed up clearly on CT or MRI scans.

The Euclidean distance transform method is widely applied in many fields, such as image processing, computer vision, graphics, and computation geometry [14]–[17]. In the current study, it was applied to calculate geodesic distance with the objective of improving speed. The N-dimensional Euclidean distance transform is used to determine the Euclidean distance of each gridding point from its nearest feature point, and consequently constructs an N-dimensional Euclidean distance field. Most of the existing methods attain the Euclidean distance field by first computing a Voronoi diagram [18], [19], in which the information for the feature point closest to each gridding point is stored. With the development of parallel hardware (i.e. a graphics processing unit [GPU]), the implementation of the Euclidean distance transform can also be transplanted to the GPU to achieve much higher computational performance. However, it is difficult to use the existing 3D Euclidean distance transform method for the optimization of radiotherapy target volume because this method fails to cope with 3D barriers. In this study, we realized a fast 3D Euclidean distance transform by extending the existing 2D Euclidean distance and used it to compute the 3D Voronoi diagram of the glioma dataset, and the shortest Euclidean distance from each gridding point in space to the glioma. Considering the influence of such barriers as the cerebral falx during target volume growth, we could swiftly solve the intersections between each segment and the barrier mesh by constructing a geometrical representation of the barriers based on the octree structure. The spatial distance field of the sheltered area was recalculated several times during iterative growth and the optimized CTV was finally obtained. It was demonstrated in the present study that our novel method of integrating 3D geometry computation with the 3D visualization analysis technique could assuredly be applied to the clinical treatment planning system.

## Conclusions

The new method we have developed to generate the CTV from the GTV using the geodesic distance calculation automatically crosses natural barriers. It is feasible for use in glioma radiotherapy and has demonstrated not only a considerable reduction in the time required to generate the CTV, but also an improvement in the accuracy of delineation. It should be admitted that only there was one patient included in this preliminary study since our objective was to find a proof of the principle method. We are now doing our further study applying this new method in much more complicated circumstances involving more anatomical barriers and different tumor sites. We also plan to evaluate if there is a dosimetric advantage regarding the target volume and critical target organs when this new approach is integrated into TPS software.

Apart from anatomical barriers, white matter fiber orientation and tumor growth kinetics should also be taken into account when performing CTV expansion. Therefore, in our future study we will also evaluate DTI combined with visualization technology, formulate a corresponding mathematical model, and calculate the 3D geometric fields using iso-surface extraction techniques. The current study provides a theoretical base regarding new criteria for target definition in postoperative radiotherapy of gliomas.
